# Global Longitudinal Strain score predicts subclinical cardiac involvement of multiple sclerosis patients

**DOI:** 10.1007/s10072-025-08041-w

**Published:** 2025-02-15

**Authors:** Fidel Demir, Mehmet Özbek, Mehmet Ata Akıl, Eşref Akıl

**Affiliations:** 1 Department of Neurology, Selahaddin Eyyubi State Hospital, Diyarbakır, Turkey; 2 Department of Cardiology, Faculty of Medicine, Dicle University, Diyarbakır, Turkey; 3 Department of Neurology, Faculty of Medicine, Dicle University, Diyarbakır, Turkey

**Keywords:** GLS, EDSS, Multiple sclerosis, Echocardiography

## Abstract

**Background:**

People with multiple sclerosis (MS) have a higher risk of cardiovascular disease than the general population, but the data are limited. Evaluation with strain echocardiography, a new echocardiographic method, can provide more objective data to evaluate global and segmental left ventricular systolic functions. Left ventricular Global Longitudinal Strain (GLS) may be useful in demonstrating subclinical myocardial dysfunction in MS, therefore we planned such a study. We aim to evaluate LV functions with GLS obtained with basal tissue doppler in people with MS.

**Material and methods:**

A comparison of the demographic laboratory and echocardiographic findings of the multiple sclerosis patients with strain echocardiography records registered in our hospital and the control group with similar age and gender was performed. 80 RRMS patients and 65 control group were compared. Those with another chronic disease, those who received exacerbation treatment within the last month, those outside the age range of 18–65, and other forms of progressive MS were excluded from the study.

**Results:**

GLS scores was significantly lower in the MS group(-17.05 ± 1.33 *vs.*18.99 ± 1.08, ***p* < *0.001*)**. The optimal GLS score predicted poor LV functional status in people with MS with high EDSS scores with cut-off value ≤ 17.10, sensitivity of 73%, specificity of 58% [AUC: 0.652 95% CI, (0.531–0.773), **p = 0.023**]. It was observed that as the EDSS score increased, that is, in the presence of worse clinical condition, the GLS score decreased (r = -0.245, **p = 0.003**).

**Conclusions:**

We think that strain echocardiography may be useful in demonstrating subclinical myocardial damage in people with MS. We found that as the EDSS score, that is, the severity of the disease, increases, the subclinical effect on cardiac functions increases.

## Introductıon

Multiple Sclerosis (MS) is the most common demyelinating disease affecting the Central Nervous System (CNS), especially in young people [1]. MS is a chronic disease with demyelination, which is involved in different localizations, possibly through genetic and environmental interactions [2]. People with MS have a higher risk of cardiovascular disease than the general population, but the data are limited [3]. Although the anomalies that MS produces in different organs are known, the effects on the cardiovascular system are not well known. Few studies in the literature examine right ventricular (RV) and left ventricular (LV) functions, and the results of these studies have been seen as contradictory [4, 5]. Abnormalities in parasympathetic or sympathetic cardiovascular tests are common in people with MS^6^.

Classical echocardiographic methods provide very subjective and insufficient information in determining ventricular function. In classical ecocardiography, left ventricular ejection fraction (LV EF) is measured and routine cardiac evaluation is performed. In the early phase, left ventricular EF is kept within normal limits with radial and circumferential compensation. In this case, classical echocardiographic does not detect early cardiac pathology. In early myocardial disease, longitudinal function is reduced. In myocardial pathologies with preserved LV EF, longitudinal function can be evaluated by strain ecocardiography [7]. Evaluation with strain echocardiography, a new echocardiographic method, can provide more objective data to evaluate global and segmental left ventricular systolic functions. It has been shown that left ventricular Global Longitudinal Strain(GLS) echocardiography method provide quantitative information about regional myocardial deformations and global functions [8]. Further investigation of cardiac involvement in multiple sclerosis with new echocardiographic methods is needed. In previous study, GLS score in other autoimmune disease was found to be lower than in the normal population [9].

We aim to evaluate LV functions with GLS obtained with basal tissue doppler in people with MS. We also wanted to investigate the relationship between the Expanded Disability Status Scale (EDSS) score, which is the severity of MS disease, and the GLS score. GLS score may be useful in demonstrating subclinical myocardial dysfunction in people with MS, therefore we planned such a study.

## Methods

### Ethics approval statement

This study was approved by the Dicle University Clinical Research Ethics Committee (Date: 17.04.2024, No: 152). Written informed consent was obtained from all study participants.

### Study population and design

This study was conducted with the contributions of our university, Faculty of Medicine, Department of Neurology,by comparing all MS patients whose strain echocardiography recordings could be evaluated with the recordings of the echocardiography device in the Department of Cardiology between January 2022 and January 2023, and the control group with similar characteristics.

80 RRMS patients with 2010 McDonald's diagnostic criteria who were followed in the Neurology Department during the study were included. The control group consisted of 65 healthy volunteers, selected from among healthcare workers and medical students in the hospital, without known cardiological and systemic diseases. Patients and control groups were included in the detailed history, including their cardiologic and neurological backgrounds. Patients with coronary artery disease history, electrocardiographic changes such as myocardial ischemia, known LV dysfunction, heart failure, hypertrophic cardiomyopathy, atrial fibrillation, diabetes mellitus, hypertension, chronic obstructive pulmonary disease and asthma, pregnancy, patients younger than 18 years and older than 65 years, patients with mild or severe valve anomalies, chronic renal insufficiency and insufficient echogenicity were excluded from the study.

Of the 80 RRMS patients, 29 were using glatiramer acetate, 14 were using fingolimod, 10 were using ocrelizumab, 9 were using interferon beta, 2 were using teriflunamide, 1 was using rituximab, 3 were using cladribine, and 12 patients were being followed up without using medication.

Visual, brain stem, pyramidal, cerebellar, sensory, bowel and bladder and cerebral functions are evaluated in EDSS score calculation. It reflects the severity of the clinical condition of the patients. The age of the patients, the number of MS attacks in the last year, EDSS scores and T2, FLAIR sequence MRI lesions (periventricular, cortical-juxta cortical, infratentorial, spinal) parameters were determined by a qualified neurologist.

### LV strain measurements

During routine controls in the neurology clinic, routine blood parameters of peripheral blood were studied. Echocardiographic images of the patients included in the study were taken at the end of expiration using a Vivid 6 (GE Medical Systems, USA) echocardiography device in the left lateral position, and echocardiographic studies were based on the guidelines of ASE [10]. GLS measurement technique for optimum measurements, myocardial walls should be clearly defined and the myocardium and surrounding structures should be distinguished from each other. Since the resulting myocardial velocities and deformation curves vary at different section velocities, the transducer axis should be parallel to the myocardial wall for a better result [11]. GLS was calculated from high frame rate (> 50 frames/sec) apical views (four, two, and three chambers) [12]. By following the endocardial borders in one end-systolic frame, myocardial speckles were automatically monitored in subsequent frames. Adequate tracking verified and manually corrected. Left ventricular end-diastolic diameter (LVEDD), left ventricular end-systolic diameter (LVESD), ejection fraction (%EF = [stroke volume/end-diastolic volume] × 100), and GLS values were used to assess systolic function [13].The main wave peak systolic strain occurring in the strain curves and this wave are examined in the evaluation. In order to avoid bias in the evaluations, echocardiographic records of the entire control group were taken first. Echocardiographic evaluations of all registered patients were performed by two different researchers using the offline EchoPAC software of the Vivid 6 (GE Medical Systems, USA) echocardiography device.

### Statistical analysis

For the statistical analysis, the SPSS version 23 (IBM Corp., Armonk, NY, USA) program was used. The normal distribution suitability of the variables was examined by visual and analytical methods. Descriptive statistics for normal distributive data were expressed as mean ± standard deviation, as a percentage for nominal data. A comparison of two or more group ratios was done by chi-square test. Comparison of the average of the two groups, the parametric test was performed with an independent-samples-t-test when assumptions were made. Mann–Whitney U test was used when parametric test assumptions were not provided. In the study, the results were evaluated at a 95% confidence interval, significance at p < 0.05 significance level, and *p* < *0.05* was considered statistically significant between the groups. In order to assess the reliability between operators coefficient of variation (CV) analysis were performed. The CV values compared with Paired Samples T test in Table 1. No significant difference was observed between the two operator for the coefficient of variance and the GLS.

**Table 1:** Coefficient of variation assesment between operators

Parameters	Operator 1	Operator 2	*P value*
Global longitudinal strain	18,59 ± 1,26	18,65 ± 1,52	0.694
Coefficient of variance	6.7%	8.1%	

## Results

In this study, 80 people with MS (25 males and 55 females) and a control group of 65 (21 males and 44 females) were compared. Laboratory parameters, echocardiographic measurements and demographic parameters of MS and control groups are shown in Table 2. While time since first MS symptom of our RRMS patients was 9.1 ± 3.6 years, time since diagnosis MS years of the disease was 7.2 ± 3.5 years. Serum albumin level was found to be significantly lower in the MS group in terms of biochemical parameters. There were no significant differences between the two groups in terms of other biochemical parameters. Serum lymphocyte count was found to be significantly lower in the MS group in terms of hemogram parameters of MS and control groups.

**Table 2:** Demographic Characteristics, echocardiographic measurements and laboratory characteristics of MS and control groups

Variables	MS Patients (n = 80)	Control Group(n = 65)	P value
Age (Year)	36,9 ± 9,2	35,9 ± 8,7	0.543
Gender (male)(%)	25(31)	21(32)	0.516
Albumin(g/dl)	3,97 ± 0,4	4,14 ± 0,5	**0.018**
Glucose (mg/dL)	93,1 ± 13	95,2 ± 12,2	0.309
WBC (K/uL)	6631 ± 1921	7235 ± 1890	0.060
Hgb (g/dL)	13,3 ± 2,1	13,8 ± 1,9	0.128
Hct (%)	41,1 ± 5,7	42,7 ± 5,1	0.073
PLT (K/uL)	254 ± 57	279 ± 57	0.343
Lymphocyte (Null)	1653 ± 723	2161 ± 663	** < 0.001**
Neutrophils (Null)	4195 ± 1479	4227 ± 1582	0.900
Triglycerides (mg/dL)	125 ± 52	142 ± 75	0.103
Total Cholesterol (mg/dL)	180 ± 28	180 ± 31	0.928
HDL(mg/dL)	47 ± 9	46 ± 8	0.727
LDL (mg/dL)	108 ± 24	105 ± 24	0.527
LVEF(%)	62,7 ± 4,1	63,2 ± 3,7	0.468
Deceleration time(msec)	170,9 ± 28,2	173,2 ± 19,3	0.591
E wave velocity (cm/sec)	80,2 ± 13,8	84,7 ± 11,3	**0.034**
A wave velocity (cm/sec)	64,1 ± 12,9	63,7 ± 9,3	0.842
E / A Ratio	1,28 ± 0,25	1,36 ± 0,26	0.069
GLS score(%)	17,05 ± 1,33	18,99 ± 1,08	** < 0.001**
Time since first MS symptom, years (mean ± SD)	9,1 ± 3,6		
Time since diagnosis MS years (mean ± SD)	7,2 ± 3,5		
EDSS score	2,48 ± 1,28		

EDSS-Expanded Disability Status Scale, GLS- Global Longitudinal Strain,Hct- hematocrit, HDL- High Density Lipoprotein,

Hgb- Hemoglobulin, LDL- Low Density Lipoprotein, LVEF- Left Ventricular Ejection Fraction,

PLT- Platelet count, WBC- White Blood Cell

**Fig. 1:**
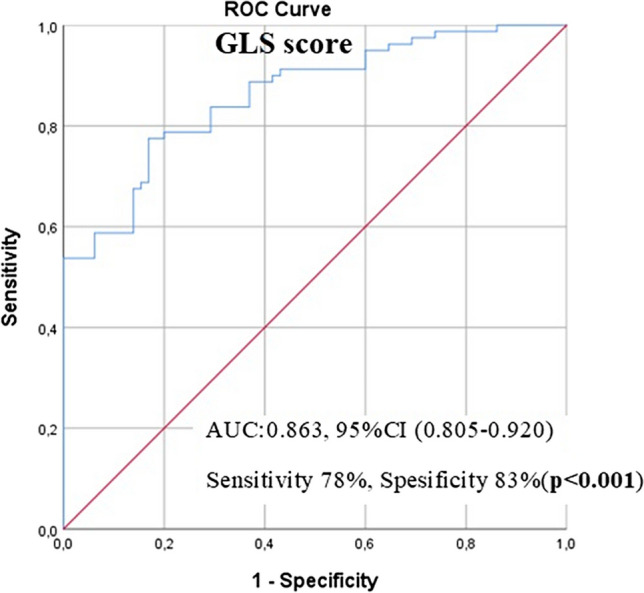
Optimum GLS score cut-off value for the estimation of echocardiography scores of people with MS and the control group was determined using receiver operating characteristic (ROC) curve analysis. AUC: Area under the curve, CI: Confident interval

E wave velocity was significantly lower in the MS group than in control groups to evaluate diastolic functions (80.2 ± 13.8 versus 84.7 ± 11.3, ***p* = *0.034***). GLS scores was significantly lower in the MS group (−17.05 ± 1.33 *vs.*18.99 ± 1.08, ***p* < *0.001*)**. We divided MS patients into two groups according to the median EDSS score. We investigated the differences in GLS scores between the two groups. We have given the optimum differential GLS score in comparison of the groups formed according to the median 2 EDSS score, using receiver operating characteristic (ROC) curve analysis in Fig. 1. The optimal GLS score predicted poor LV functional status in people with MS with high EDSS scores with cut-off value ≤ 17.10, sensitivity of 73%, specificity of 58% [AUC: 0.652 95% CI, (0.531–0.773), p = 0.023].

In our MS group consisting of 80 patients, the median EDSS score was calculated as 2.00 The Spearman correlation analysis of the GLS score and other variables in the MS group is given in Table 3. There seems to be a weak correlation between GLS and EDSS score (r = −0.245, **p = 0.003**). It was observed that as the EDSS score increased, that is, in the presence of worse clinical condition, the GLS score decreased.

**Table 3:** Spearman Correlation Analysis for Multiple Sclerosis Patients

	GLS	
	r	*p*
EDSS score	−0.245	***0.003***
Age	0.136	0.079
Gender (male)	−0.026	0.783
MS attack count in last year	−0.184	***0.045***
Lesion count in MRI scan	−0.185	***0.041***

## Discussion

MS patients are in the age group with a lower risk of cardiovascular disease. In assessing cardiac function in this way, we planned to explore the impact of MS as much as possible. As the main findings of our study: 1) We found a significantly lower GLS score in people with MS compared to the age- and gender-matched control group, 2) We found a correlation between the GLS score and the EDSS score, 3) We found that strain imaging can be effective in early detection of the increased incidence of cardiovascular disease in MS patients. 4) And as the most important result, we found that as the EDSS score, that is, the severity of the disease, increases, the subclinical effect on cardiac functions increases.

Our study, which shows the comparison of groups with no cardiac findings yet, is very important in terms of demonstrating the value of strain echocardiography, a new echocardiographic evaluation tool. A significant difference between the MS patients and the control group, and a significant difference between the groups after grouping according to the EDSS score are very important results. Considering the immunological, functional and clinical differences between the groups, it is observed that cardiac involvement is determined by internal and external factors. We expect that interventions that will improve the current situation will reduce the level of cardiac involvement.

A study was also conducted comparing people with MS without cardiac signs and symptoms and a healthy control group. LVEF was significantly lower in people with MS^14^. In studies conducted in recent years, no statistically significant difference was observed between people with MS and control groups in terms of LVEF [15, 16]. In another study MS and the control group were evaluated with radionuclide angiography, which can be considered the gold standard for the detection of EF. There was a statistically significant decrease in RVEF and LVEF values in people with MS compared to the control group [17]. While the mean LVEF values calculated by standard echocardiography in our study did not differ significantly between the two groups, the significant difference between the GLS scores indicates that this evaluation method may be significant in terms of showing cardiac involvement in the early period.

In a well-designed study, on strain echocardiography, there was a significant decrease in GLS values in infarcted segments compared with normal subjects, indicating a strong correlation with the 16-segment wall motion score index. In this study, it was reported that these new myocardial assessment methods were effective in quantitatively identifying normal and chronic infarcted tissues [18]. GLS measurements provide important information about regional functions as well as global functions [19].

Evaluation of wall motion disorder with echocardiography is largely user dependent. In our study, GLS values were lower in the patient group than in the control group. This difference was statistically significant. In conclusion; It has been shown that MS may impair systolic functions by increasing the frequency of pre-detected ischemic heart disease or by causing cardiovascular disease secondary to autonomic system dysfunction. Our study proved that, regardless of this etiology, increased cardiovascular disease involvement can be demonstrated even in the early period with strain echocardiography compared to the normal population.

In a recent and very important study, it was emphasized that cardiovascular risk in MS patients is increased by genetic factors and the importance of active monitoring and prevention of cardiovascular risk in the fight against cardiovascular comorbidities in MS patients [20]. We think that with the reduced GLS score in our study compared with the control group, we found that subclinical cardiovascular involvement can be demonstrated in the early period. we also think that the weak correlation with the EDSS score is very important even in the present MS group with little cardiac involvement. It is obvious that cardiovascular involvement may affect the severity of the disease, but it is thought that the severity of the disease may also predict the development of cardiovascular disease. For this reason, we think that cardiological follow-up is very important in terms of all kinds of prognostic effects in MS patients, as the most important result of our study. In this follow-up, we suggest that the use of strain imaging technique, which can be considered as an advanced imaging method with the available evidence, should be increased.

## Conclusion

We suggest that the use of strain imaging technique, which can be considered as an advanced imaging method with the available evidence, should be increased. We think that strain echocardiography may be useful in demonstrating subclinical myocardial damage in people with MS. We think that the evaluation of patients with any cardiovascular follow-up method, which is not harmful for use in MS patients, can provide serious prognostic data. We also believe that more comprehensive studies are needed on this subject.

## Limitations

Although our study is the first in the literature to investigate myocardial functions in people with MS with Strain echocardiographic imaging methods, it has some limitations. The fact that it is a single-center study can be considered a limitation. The small number of patients and not knowing the disease activity level can be considered as a limitation. We retrospectively screened MS patients who were followed up in our clinic and whose echocardiographic images were recorded, and recorded all reliable data of the patients retrospectively. We identified it as a limitation that very few data can be collected about the clinic of the patients.

## Data Availability

Data available on request due to privacy/ethical restrictions.
